# Risk of Delayed Post‐polypectomy Bleeding in Patients With Antithrombotic Therapy

**DOI:** 10.1002/deo2.70401

**Published:** 2026-08-11

**Authors:** Rumiko Tsuboi, Satohiro Matsumoto, Makiko Mieno, Shizukiyo Ishikawa, Yurika Imai, Mina Morino, Keita Matsumoto, Yudai Koito, Hitomi Kashima, Takaya Miura, Yuko Takahashi, Takehiro Ishii, Shuhei Yoshikawa, Haruka Otake, Masanari Sekine, Takeshi Uehara, Takeharu Asano, Hiroyuki Miyatani, Hirosato Mashima

**Affiliations:** ^1^ Department of Gastroenterology Jichi Medical University Saitama Medical Center Saitama Japan; ^2^ Center For Information Jichi Medical University Tochigi Japan

**Keywords:** antithrombotic agent, colonic polyp, delayed hemorrhage, polypectomy, risk factors

## Abstract

**Objectives:**

Recent changes in the perioperative management of antithrombotic therapy (ATT), including the discontinuation of routine heparin bridging and the increased use of cold snare polypectomy, may reduce delayed post‐polypectomy bleeding (DPPB) rates. However, few recent studies have evaluated these changes. We compared DPPB rates between patients with and without ATT and explored factors associated with bleeding.

**Methods:**

We conducted a retrospective cohort study of adult patients who underwent endoscopic resection of colorectal polyps ≤15 mm between April 2021 and October 2023. DPPB was defined as hematochezia or a hemoglobin drop ≥2.0 g/dL requiring endoscopic hemostasis. Univariable analyses were performed to identify factors associated with DPPB, and exploratory multivariable analyses were conducted.

**Results:**

Among 2136 patients with 7025 polyps, 523 patients (1874 polyps) were receiving ATT. The DPPB rates per patient (1.91% vs. 0.25%) and per lesion (0.69% vs. 0.08%) were significantly higher in the ATT group (*p* < 0.001). In univariable analyses, ATT use (odds ratio [OR] 7.84), ≥3 resected lesions (OR 5.13), and hypertension (OR 3.95) were significant risk factors in the per‐patient analysis, while ATT use (OR 8.73), lesion size >10 mm (OR 4.51), and clipping (OR 5.20) were significant in the per‐lesion analysis. Among ATT types, warfarin users had the highest DPPB rates.

**Conclusions:**

Colorectal polypectomy in patients receiving ATT is associated with an increased risk of DPPB. Particular caution is warranted in patients receiving warfarin.

**Trial Registration:**

N/A

## Graphical abstract

Antithrombotic therapy is associated with an increased risk of delayed post‐polypectomy bleeding after endoscopic resection of colorectal polyps ≤15 mm. Despite this increased risk, overall bleeding rates remain low in both per‐patient and per‐lesion analyses.

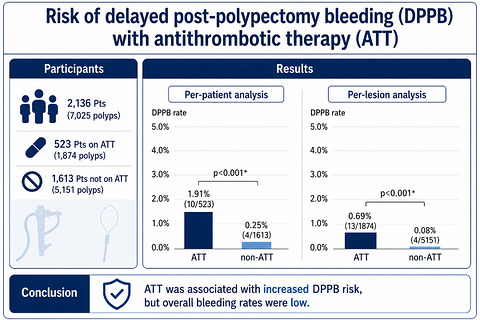

## 1 Introduction

Endoscopic colorectal polypectomy reduces the incidence of colorectal cancer [1] and related mortality [2] by removing adenomatous polyps. The use of antithrombotic agents has increased owing to the growing burden of cardiovascular and cerebrovascular diseases, expansion of preventive medicine, and widespread use of direct oral anticoagulants (DOACs) [3‐–5].

Delayed post‐polypectomy bleeding (DPPB) occurs in approximately 1.5% of cases [6], with the rate increasing to 13.7% in patients receiving anticoagulants such as DOACs and warfarin [7]. This previous study included patients who underwent heparin bridging, which has been shown to increase the risk of DPPB [8, 9, 10]. Accordingly, the 2017 appendix to the Japan Gastroenterological Endoscopy Society (JGES) antithrombotic guidelines no longer recommended routine heparin bridging in patients receiving anticoagulants [11]. Cold snare polypectomy (CSP) has been associated with low DPPB rates of 1.64% and 0.37% in patients with and without antithrombotic agents, respectively [12]. In contrast, the DPPB rates after endoscopic mucosal resection (EMR) have been reported to range from 0.6% to 6.2% [13].

The discontinuation of heparin bridging and widespread adoption of CSP may have reduced DPPB rates, even among patients receiving antithrombotic therapy (ATT). Recent studies have focused on individual resection techniques, particularly CSP, and data encompassing CSP, EMR, and hot snare polypectomy (HSP) remain scarce. In clinical practice, patients often have multiple polyps, and the appropriate resection technique for each lesion is determined during colonoscopy. Therefore, comprehensive data encompassing all commonly used resection techniques are needed to estimate the bleeding risk in real‐world practice. This need was further highlighted by our transition from inpatient to outpatient polypectomy for patients receiving ATT during the COVID‐19 pandemic. Although the JGES colorectal endoscopic submucosal dissection/EMR guidelines allow outpatient EMR for polyps <20 mm, no specific recommendations are provided for patients undergoing ATT [14]. Therefore, an evidence‐based assessment of bleeding risk is required.

The present study aimed to compare the incidence of DPPB between patients undergoing endoscopic colorectal polypectomy (including CSP, EMR, and HSP) with and without ATT, and to evaluate the impact of ATT on DPPB risk.

## 2 Methods

### 2.1 Study Design

This retrospective cohort study was conducted at a single center and reviewed and approved by the Clinical Research Ethics Committee of Jichi Medical University Saitama Medical Center (approval no. Rin‐S24‐040). All data were collected in May 2025.

### 2.2 Participants

We included adult patients who underwent endoscopic resection of colorectal polyps ≤15 mm at Jichi Medical University Saitama Medical Center between April 2021 and October 2023. Patients who underwent simultaneous endoscopic submucosal dissection or whose pathological results were unavailable were excluded. Informed consent was obtained using the opt‐out approach.

### 2.3 Clinical Data Assessment

We collected the following data from medical records. Patient‐related factors included age, sex, comorbidities potentially indicating ATT (cardiovascular disease, hypertension, cerebrovascular disease, peripheral vascular disease, deep vein thrombosis, diabetes mellitus, dialysis, respiratory disease, and spinal disorders), use of ATT, and number of resected lesions. Operator‐related factors included the certification status according to the JGES. Lesion‐related factors included location (proximal, cecum to transverse colon; distal, descending colon to sigmoid colon; rectum), size (measured with a snare), morphology (classified using the Paris classification: 0‐Ip [pedunculated], 0‐Is/0‐Isp [sessile], and 0‐II [non‐polypoid]), use of electrocautery (EMR or HSP), piecemeal resection, prophylactic clipping, and histopathological findings (cancerous vs. non‐cancerous).

### 2.4 Colonoscopy

Endoscopic colorectal polypectomy was performed for pre‐identified polyps or for pre‐unidentified polyps discovered during the examination. Before the procedure, the patients were confirmed to have no hematologic or coagulation abnormalities that would preclude colorectal polypectomies. Most patients had a low‐residue diet the day before colonoscopy, consumed 10 mL of sodium picosulfate orally before sleep, and consumed polyethylene glycol orally the morning of the procedure. Anticholinergic agents or glucagon were administered intramuscularly unless contraindicated, and sedatives were administered intravenously when necessary. Colonoscopy was performed using magnifying endoscopes (PCF‐H290ZI and PCF‐Q260AZI; Olympus Medical Systems).

CSP was avoided for lesions suspected of malignancy or measuring ≥10 mm. When benign lesions were suspected, the resection method was selected at the discretion of the endoscopist. The snares used were Captivator II 10 or 15 mm, Captivator 13 mm (Boston Scientific, USA), and Polypectomy Snare 15 mm (AGS MedTech, China).

Resected polyps were retrieved by suction or using forceps/nets and submitted for pathological analysis. Prophylactic clipping was performed at the endoscopist's discretion, either for immediate hemostasis or for the prevention of DPPB; in routine practice, clipping was generally performed following EMR or HSP. All patients returned for outpatient follow‐up within one month to receive the pathology results and to assess for any adverse events, including DPPB and thromboembolic events.

### 2.5 Management of Antithrombotic Agents

Antithrombotic management was based on the 2012 JGES guidelines [15] and the 2017 appendix regarding DOACs [11]. Orders were made in consultation with prescribing physicians and included the following: aspirin was either continued or withheld for 3–5 days, thienopyridines were discontinued for 5–7 days or substituted with aspirin or cilostazol, and other antiplatelets were stopped for 1 day. Warfarin was continued if the prothrombin time‐international normalized ratio (PT‐INR) was within the therapeutic range the day before or on the day of the procedure. DOACs were withheld only on the day of the procedure. Patients taking multiple agents were asked to discontinue any medications that could be discontinued. If necessary, patients were allowed to continue taking aspirin, cilostazol, or warfarin alone. The nurses confirmed and recorded the status of the antithrombotic agents on the day of the procedure. In the absence of bleeding, antithrombotic agents were resumed on the following day.

### 2.6 Outcome Measures

The primary outcome was the DPPB rate in the ATT and non‐ATT groups. Secondary outcomes included risk factors for DPPB, DPPB rates stratified by antithrombotic agent type, and DPPB rates in subgroups based on continuation or withdrawal of antithrombotic agents. Analyses stratified by the antithrombotic agent type were conducted on a per‐exposure basis in both per‐patient and per‐lesion analyses because some patients received combination ATT. DPPB was defined based on the JGES criteria [14] as bloody stool or a hemoglobin drop of ≥2.0 g/dL after polypectomy, requiring endoscopic hemostasis.

### 2.7 Statistical Methods

Categorical variables were analyzed using the chi‐square or Fisher's exact test. Continuous variables with a normal distribution (age and lesion size) were compared using the t‐test. The number of resected lesions, which were non‐normally distributed, was analyzed using the Mann‐Whitney U test. Univariable and multivariable analyses were performed to identify risk factors for DPPB. Variables with a *p*‐value <0.05 in the univariable analysis were considered for inclusion in the multivariable models, along with clinically relevant variables based on prior evidence. For the per‐patient analysis, a multivariable logistic regression model using the Firth procedure was performed, adjusting for ATT, hypertension, and number of polyps resected ≥3. Cardiovascular disease, although previously reported as a risk factor for DPPB [9], was not included in the multivariable model because of its strong correlation with ATT. For the per‐lesion analysis, generalized estimating equations (GEE) were used to account for within‐patient clustering due to multiple lesions per patient. The final multivariable model included ATT, lesion size >10 mm, and electrocautery. Clipping was not included due to its correlation with electrocautery and to maintain model parsimony. All statistical analyses were performed using EZR version 1.67 [16], with R version 4.3.3 used for the GEE and Firth procedure. Statistical significance was defined as a two‐sided *p*‐value of <0.05.

## 3 Results

### 3.1 Patient Flow

**FIGURE 1:**
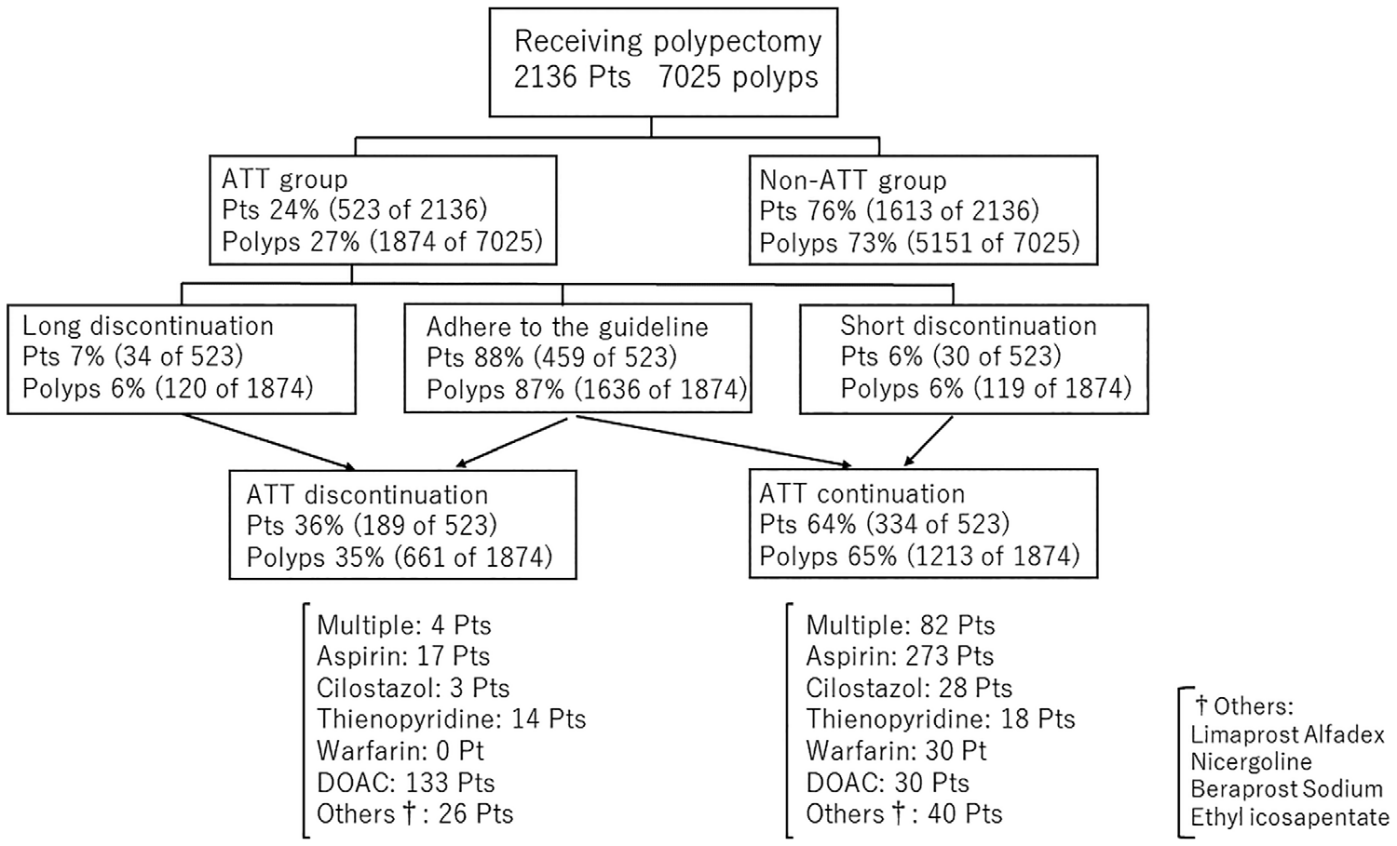
Study flow diagram. Pts: patients; ATT: antithrombotic therapy.

A total of 2136 patients with 7025 polyps underwent colorectal polypectomy. Namely, 523 patients (24%) with 1874 polyps (27%) were in the ATT group, and 1613 patients (76%) with 5151 polyps (73%) were in the non‐ATT group. For subgroup analysis, patients in the ATT group were classified according to adherence to the JGES guidelines; those with “longer‐than‐guideline” withdrawal and “guideline‐compliant” withdrawal were categorized as the withdrawal group, and those with “shorter than guideline” withdrawal or “continued antithrombotic agents as per guideline” were categorized as the continuation group. The withdrawal group included 189 patients (36%) and 661 polyps (35%), while the continuation group included 334 patients (64%) and 1213 polyps (65%) (Figure 1).

### 3.2 Background Characteristics of Patients and Polyps

The ATT group had a significantly higher proportion of males, older age, and more procedures performed by non‐specialist endoscopists. Among the polyps, no significant differences between the groups were seen in the mean lesion size, proportion of pedunculated (Ip) polyps, rate of CSP, or incidence of carcinoma. However, proximal polyp location and prophylactic clipping were significantly more frequent in the ATT group (Tables 1 and 2).

**TABLE 1:** Characteristics of the study participants in antithrombotic therapy and non‐antithrombotic therapy groups.

Patient	ATT group *N*=523	Non‐ATT group *N*=1613	*p*‐Value
Male	419 (80.1%)	1035 (64.2%)	<0.001*
Age, Mean ± SD	74.3 ± 7.7	67.5 ± 11.5	<0.001*
Comorbidities, *n* (%)			
Cardiovascular disease	368 (70.4%)	126 (7.8%)	<0.001*
Hypertension	354 (67.7%)	678 (42.0%)	<0.001*
Cerebrovascular disease	139 (26.6%)	41 (2.5%)	<0.001*
Peripheral vascular disease	24 (4.6%)	6 (0.4%)	<0.001*
Deep vein thrombosis	8 (1.5%)	9 (0.6%)	0.044*
Diabetes mellitus	186 (35.6%)	297 (18.4%)	<0.001*
Dialysis	42 (8.0%)	33 (2.0%)	<0.001*
Respiratory disease	50 (9.6%)	100 (6.2%)	0.012*
Spinal disorders	12 (2.3%)	19 (1.2%)	0.089
No. of polyps resected, Median(range)	3 (1‐13)	3 (1‐16)	<0.001*
Specialist‐performed	330 (63.1%)	1122 (69.6%)	0.007*

ATT: antithrombotic therapy; SD: standard deviation.

*p < 0.05.

Statistical tests: Chi‐square test (male, cardiovascular disease, hypertension, cerebrovascular disease, peripheral vascular disease, diabetes mellitus, dialysis, respiratory disease, spinal disorders, and specialist‐performed); *t*‐tests (age); Fisher's exact test (deep vein thrombosis); Mann‐Whitney U test (no. of polyps resected).

**TABLE 2:** Characteristics of polyps in each group.

Polyp	ATT group *N* = 1874	Non‐ATT group *N* = 5151	*p*‐Value
Diameter (mm), Mean ± SD	5.46 ± 2.74	5.46 ± 2.80	0.958
Morphology, *n* (%)			
Non‐polypoid (type II)	430 (22.9%)	1518 (29.5%)	<0.001^*^
Sessile (0‐Is, Isp)	1385 (73.9%)	3476 (67.5%)	<0.001^*^
Pedunculated (Ip)	59 (3.1%)	157 (3.0%)	0.891
Location, *n* (%)			
Proximal colon	1187 (63.3%)	2965 (57.6%)	<0.001^*^
Distal colon	687 (36.7%)	2186 (42.4%)	
Rectum	110 (5.9%)	407 (7.9%)	
Polypectomy method			
EMR, HSP	563 (30.0%)	1471 (28.6%)	0.236
CSP	1311 (70.0%)	3680 (71.4%)	
Piecemeal resection	26 (1.4%)	100 (1.9%)	0.148
Clipping	1349 (72.0%)	2514 (48.8%)	<0.001^*^
Histopathological findings			
Cancerous	67 (3.6%)	173 (3.4%)	0.656
Non‐cancerous	1807 (96.4%)	4897 (96.6%)	

ATT: antithrombotic therapy; CSP: cold snare polypectomy; EMR: endoscopic mucosal resection; HSP: hot snare polypectomy; SD: standard deviation.

* *p* < 0.05.

Statistical tests: t‐tests (diameter) and Chi‐square test (morphology, location, polypectomy method, piecemeal resection, clipping, and histopathological findings).

### 3.3 Delayed Post‐polypectomy Bleeding Rates

**FIGURE 2:**
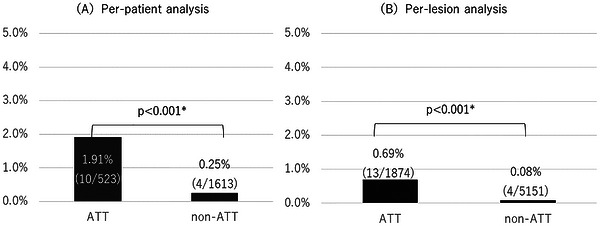
Delayed post‐polypectomy bleeding rates by ATT. (A) Per‐patient analysis. (B) Per‐lesion analysis. ATT: antithrombotic therapy. *p < 0.05 by Fisher's exact test.

The per‐patient DPPB rate was significantly higher in the ATT group (1.91%, 10/523) than in the non‐AT group (0.25%, 4/1613). Similarly, a per‐lesion analysis showed a DPPB rate of 0.69% (13/1874) in the ATT group versus 0.08% (4/5151) in the non‐ATT group (*p* < 0.001 for both comparisons) (Figure 2A,B).

### 3.4 Risk Factors for Delayed Hemorrhage

In the per‐patient univariable analysis, ATT, hypertension, and ≥3 resected lesions were significantly associated with DPPB. Given the limited number of bleeding events, multivariable analyses were considered exploratory. In a multivariable analysis, ATT and ≥3 resected lesions were associated with DPPB (Table 3); however, these findings should be interpreted cautiously. In the per‐lesion univariable analysis, ATT, lesion size >10 mm, and prophylactic clipping were significantly associated with DPPB. Electrocautery‐based procedures were not significantly associated with DPPB, although the DPPB rate tended to be higher with EMR/HSP than with CSP (0.44% [9/2034 lesions] vs. 0.16% [8/4991 lesions], *p* = 0.056). In a multivariable GEE analysis, ATT was associated with DPPB, whereas lesion size >10 mm and EMR/HSP were not (Table 3).

**TABLE 3:** Univariate and multivariate analyses of risk factors for delayed post‐polypectomy bleeding.

		Univariate		Multivariate	
	Reference	OR (95%CI)	*p*‐Value	OR (95%CI)	*p*‐Value
per‐patient analysis					
Age ≧75	Age <75	1.80 (0.63–5.15)	0.273		
Male	Female	2.83 (0.63–12.7)	0.174		
ATT	Non‐ATT	7.84 (2.45–25.1)	<0.001^*^	5.51 (1.86–19.2)	0.002*
Hypertension	None	3.95 (1.10–14.2)	0.035*	2.16 (0.68–8.73)	0.199
No. of polyps resected ≧3	<3	5.13 (1.15–23.0)	0.033*	3.57 (1.06–18.4)	0.039*
Specialist‐performed	None	1.60 (0.55–4.62)	0.388		
per‐polyp analysis					
ATT	Non‐ATT	8.73 (2.60–29.3)	<0.001^*^	8.94 (2.62–30.5)	<0.001^*^
Size >10 mm	≦10 mm	4.51 (1.41–14.4)	0.011^*^	2.79 (0.74–10.6)	0.13
Rectum	Cecum to Sigmoid colon	2.85 (0.75–10.8)	0.124		
Proximal colon	Distal colon	0.56 (0.22–1.45)	0.235		
Polypoid type	Non‐polypoid	0.52 (0.18–1.55)	0.243		
EMR or HSP	CSP	2.74(0.93–8.08)	0.067	2.07 (0.64–6.67)	0.224
Clipping	None	5.20(1.23–21.9)	0.025*		
Malignant	Non‐malignant	3.17(0.57–17.7)	0.188		

ATT: antithrombotic therapy; CI: confidence interval; CSP: cold snare polypectomy; EMR: endoscopic mucosal resection; HSP: hot snare polypectomy; OR: odds ratio. Given the limited number of events, the multivariate analyses were considered exploratory. Variables with a p‐value <0.05 in the univariate analysis were considered for inclusion in the multivariate model.

For the per‐patient analysis, a multivariate logistic regression model using the Firth procedure was performed, adjusting for use of antithrombotic agents, hypertension, and ≥3 resected lesions.

For the per‐lesion analysis, generalized estimating equations (GEE) were used. Covariates included use of antithrombotic agents, lesion size >10 mm, and electrocautery; clipping was not included due to its correlation with electrocautery and to maintain model parsimony.

* *p* < 0.05

### 3.5 Subgroup Analysis

In a subgroup analysis of patients receiving ATT, no significant difference in the incidence of DPPB was observed between single and multiple antithrombotic therapies in either the per‐patient analysis (1.60% [7/437] vs. 3.49% [3/86], *p* = 0.217) or per‐lesion analysis (0.65% [10/1543 lesions] vs. 0.91% [3/331 lesions], *p* = 0.712).

Among various antithrombotic agents, patients receiving warfarin had a significantly higher incidence of DPPB than those receiving other medications. In the per‐patient, per‐exposure analysis, the DPPB rate in warfarin users was 10% (3/30 exposures, *p* = 0.016), and in the per‐lesion, per‐exposure analysis, it was 3.4% (4/116 lesion‐level exposures, *p* = 0.006) (Tables 4 and 5). The median PT‐INR in patients with bleeding was 2.61 (range: 2.51–2.69), whereas it was 2.12 (range: 1.07–3.09) in those without bleeding. Although not statistically significant (*p* = 0.053), a trend toward higher PT‐INR levels was observed in the bleeding group. Thienopyridines were discontinued or substituted with aspirin or cilostazol, and there were no cases of DPPB among thienopyridine users.

**TABLE 4:** Delayed post‐polypectomy bleeding rates stratified by antithrombotic agent type (per‐patient, per‐exposure analysis).

	Non‐bleeding, *n*	Bleeding, *n* (%)	
Per‐patient	*N* ^†^ = 599	*N* ^†^ = 13	*p*‐Value
Aspirin	284	6 (2.0%)	1
Cilostazol	31	0 (0.0%)	1
Thienopyridine	32	0 (0.0%)	1
Others^‡^	65	1 (1.5%)	1
Warfarin	27	3 (10.0%)	0.016^*^
DOAC	160	3 (1.8%)	1

DOAC: direct oral anticoagulant.

^†^ *N* represents the cumulative number of antithrombotic therapy exposures at the patient level, not individual patients. Patients receiving multiple agents were counted more than once.

^‡^ Others include limaprost alfadex, nicergoline, beraprost sodium, and ethyl icosapentate.

* *p* < 0.05 by Fisher's exact test with Bonferroni correction.

**TABLE 5:** Delayed post‐polypectomy bleeding rates stratified by antithrombotic agent type (per‐lesion, per‐exposure analysis).

Per‐lesion	Non‐bleeding, *n*	Bleeding, *n* (%)	*p*‐Value
	***N*** ^†^ ** = 2205**	***N*** ^†^ ** = 16**	
Aspirin	1032	8 (0.8%)	0.783
Cilostazol	138	0 (0.0%)	0.616
Thienopyridine	126	0 (0.0%)	1
Others^‡^	240	1 (0.4%)	1
Warfarin	112	4(3.4%)	0.006^*^
DOAC	557	3 (0.5%)	0.766

DOAC: Direct Oral Anticoagulant.

^†^ *N* represents the cumulative number of lesion–antithrombotic agent exposures, not individual lesions. Lesions in patients receiving multiple agents were counted more than once.

^‡^ Others include limaprost alfadex, nicergoline, beraprost sodium, and ethyl icosapentate.

* *p* < 0.05 by Fisher's exact test with Bonferroni correction.

**FIGURE 3:**
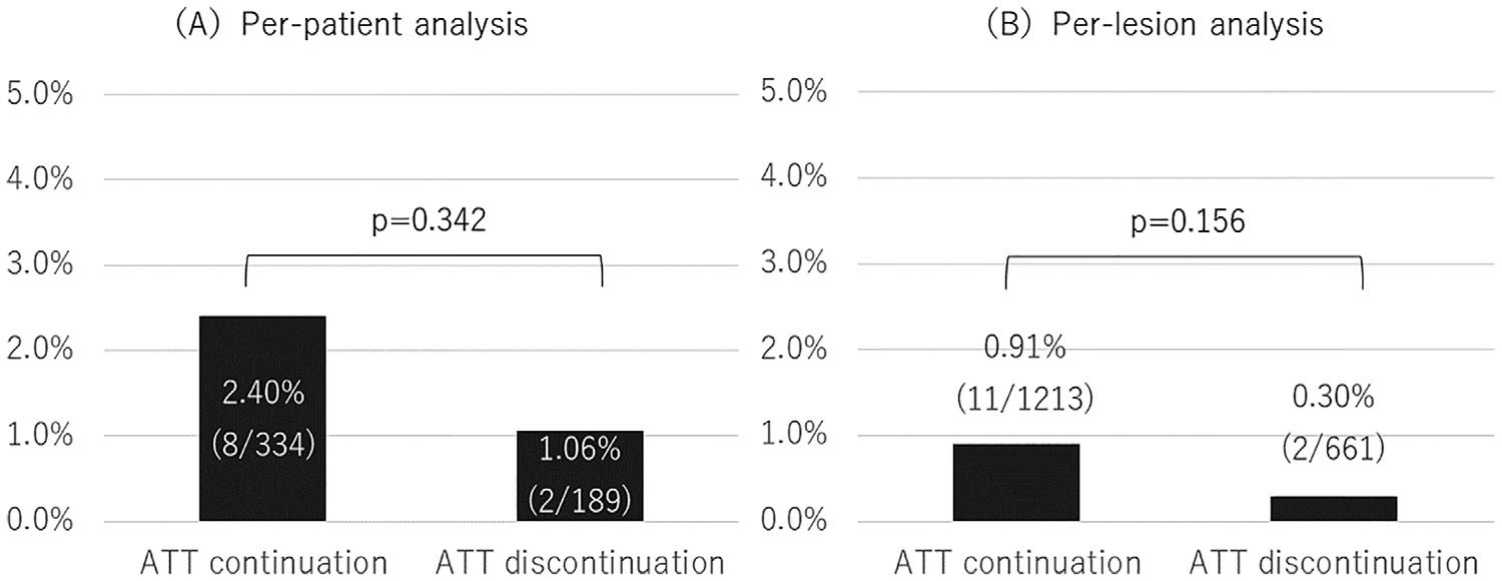
Comparison of delayed post‐polypectomy bleeding rates between continuation and discontinuation of ATT. (A) Per‐patient analysis. (B) Per‐lesion analysis. ATT: antithrombotic therapy. Fisher's exact test.

The DPPB rate was numerically higher in the continuation group than in the withdrawal group, although the difference was not statistically significant. Given the small number of bleeding events, this study was underpowered to draw definitive conclusions regarding the effect of ATT discontinuation. In the per‐patient analysis, the DPPB rates were 2.4% and 1.1% in the continuation and withdrawal groups, respectively (*p* = 0.342). Similarly, in the per‐lesion analysis, the rates were 0.91% and 0.30%, respectively (*p* = 0.156) (Figure 3A,B).

### 3.6 Bleeding Cases

**FIGURE 4:**
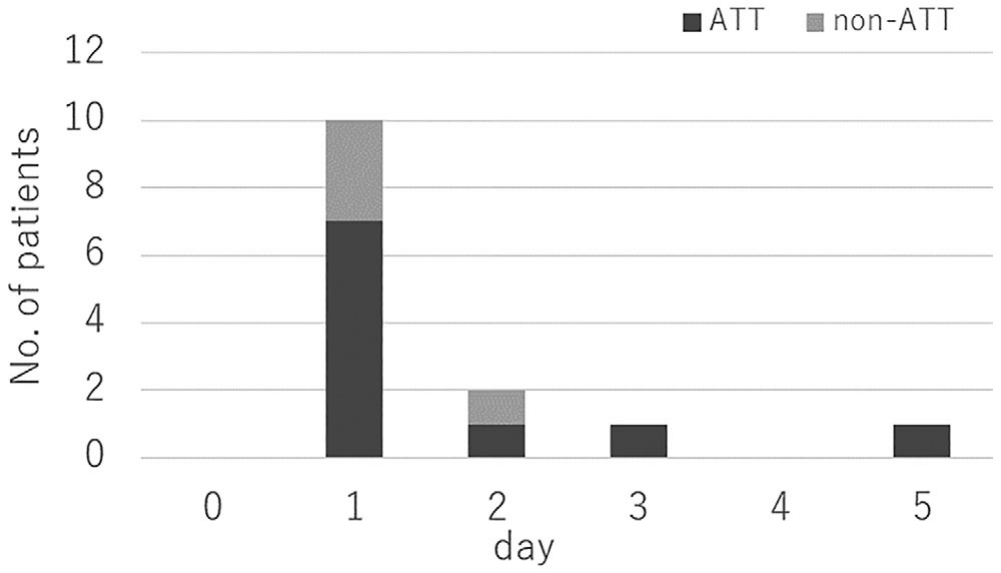
Days to post‐polypectomy bleeding. ATT: antithrombotic therapy.

A total of 17 lesions in 14 patients developed DPPB, and the details are shown in Table 6. All DPPB cases were successfully managed with endoscopic hemostasis, and no patient required blood transfusion. Most DPPB cases occurred the day after polypectomy, although bleeding was observed up to day 5 post‐procedure in the ATT group (Figure 4).

**TABLE 6:** Details of the 14 cases with delayed post‐polypectomy bleeding.

Pt. No.	Age	Sex	ATT	ATT continuation	No. of polyps resected	Location	Size (mm)	Method	Clipping	Histology	Days to hemorrhage
1	74	M	Aspirin	Continued	5	T	6	EMR	Done	Adenoma	1
2	57	M	Aspirin	Continued	3	T	5	CSP	Done	SSL	1
						T	6	CSP	Done	Adenoma	1
						D	4	CSP	Done	SSL	1
3	79	M	Aspirin	Continued	4	S	6	CSP	Done	HPP	3
4	78	M	Aspirin+EPA	EPA discontinued	4	S	13	EMR	Done	SSL	1
5	80	M	Aspirin+DOAC	DOAC discontinued	4	R	4	CSP	Not done	HPP	1
6	58	M	Aspirin+Warfarin	Aspirin discontinued	7	T	10	EMR	Done	Adenoma	1
7	75	M	Warfarin	Continued	7	T	6	CSP	Not done	Adenoma	1
8	81	F	Warfarin	Continued	4	A	6	CSP	Done	Adenoma	1
						T	7	EMR	Done	Adenoma	1
9	70	M	DOAC	Discontinued	3	T	5	EMR	Done	Adenoma	2
10	80	M	DOAC	Discontinued	4	R	12	EMR	Done	Adenoma	5
11	84	F	None		1	S	7	EMR	Done	Cancer	1
12	72	M	None		1	R	7	EMR	Done	Adenoma	2
13	73	M	None		3	S	12	EMR	Done	Cancer	1
14	55	M	None		9	D	6	CSP	Done	Adenoma	1

ATT: antithrombotic therapy; CSP: cold snare polypectomy; DOAC: direct oral anticoagulant; EMR: endoscopic mucosal resection; EPA: eicosapentaenoic acid; F: female; HPP: hyperplastic polyp; M: male; SSL: sessile serrated lesion.

A: ascending colon; D: descending colon; R: rectum; S: sigmoid colon; T: transverse colon.

## 4 Discussion

This study suggests that colorectal polypectomy performed during ATT is associated with a higher risk of DPPB. The DPPB rate was consistently higher in the ATT group in both patient‐ and lesion‐level analyses. These findings are consistent with those of previous reports [7, 17–‐19]. Because patients may have multiple polyps, previous studies have often analyzed ATT and lesion‐related factors separately rather than within a unified framework. In contrast, we applied the GEE approach to account for within‐patient clustering and evaluate the ATT and lesion‐related factors simultaneously. In univariable analyses, ATT, larger lesion size (>10 mm), and clipping were significantly associated with DPPB; however, after adjustment, lesion‐related associations were attenuated, whereas ATT remained associated with DPPB, suggesting a stronger contribution of ATT than that of individual lesion characteristics. However, given the limited number of events, the multivariable analysis should be considered exploratory, and larger studies are warranted to confirm these findings.

Previous studies have reported that electrocautery‐based procedures are associated with a higher risk of DPPB than CSP [20]. In the present study, CSP tended to be associated with a lower DPPB rate than electrocautery‐based procedures; however, this difference did not reach statistical significance, possibly due to the limited number of DPPB events in our study.

Notably, the overall incidence of DPPB in this study was lower than that reported previously [7, 10, 17]. This may reflect changes in clinical practice, including the abandonment of heparin bridging and the increased use of CSP. Although the limited number of events reduced the statistical power, it may also indicate a true reduction in DPPB incidence. In settings capable of managing bleeding complications, outpatient polypectomy in patients receiving ATT may be acceptable under antithrombotic management in accordance with the current JGES guidelines [11].

When stratified by antithrombotic agent, warfarin users had the highest DPPB rates among ATT users; however, the DPPB rates were lower than those reported in previous studies (patient‐based: 13.7% [7]; lesion‐based HSP: 6.2% [21]). Among warfarin users, PT‐INR levels tended to be higher in patients with bleeding, even within the therapeutic range. This finding raises the possibility that avoiding PT‐INR values near the upper limit of the therapeutic range may be prudent in warfarin users undergoing polypectomy, although further studies are required.

The incidence of DPPB among DOAC users was comparable to that among aspirin users and was similarly low, possibly reflecting current management strategies.

No significant difference in DPPB rates was observed between the continuation and discontinuation groups among patients receiving ATT, consistent with previous reports [22, 23]. However, DPPB rates were numerically higher in the continuation group than in the discontinuation group, and the lack of statistical significance may be attributable to the limited number of events (i.e., possible type II error). No adverse events were observed; however, in some cases, guideline‐recommended discontinuation periods were not followed due to misunderstandings by patients or physicians, suggesting a need to improve adherence to the guidelines.

This study has several limitations, including its retrospective, single‐center design, potential information bias from secondary records, and limited generalizability of the results. The small number of DPPB events also limited the statistical power and necessitated an exploratory multivariable analysis. Therefore, these findings should be interpreted with caution and validated in larger multicenter studies in the future.

Notably, this study evaluated the primary endpoint and DPPB rates stratified by antithrombotic type and continuation/discontinuation status at both the patient and lesion levels, whereas previous studies have generally been conducted at the patient level.

## 5 Conclusion

Colorectal polypectomy performed under ATT carries an increased risk of DPPB, requiring careful perioperative management. However, the incidence of DPPB appears to have decreased compared with previous reports, possibly due to the widespread use of CSP and the avoidance of heparin bridging. In this study, warfarin users remained at particularly high risk among patients receiving antithrombotic agents, warranting additional caution.

## Author Contributions

**Satohiro Matsumoto**: conceptualization, investigation, methodology, validation, writing – review and editing, formal analysis, project administration, data curation, supervision, resources, visualization, and software. **Yudai Koito**: investigation, resources, and writing – review and editing. **Rumiko Tsuboi**: conceptualization, investigation, writing – original draft, methodology, validation, visualization, writing – review and editing, software, formal analysis, project administration, supervision, data curation, and resources. **Keita Matsumoto**: investigation, resources, and writing – review and editing. **Shuhei Yoshikawa**: investigation, writing – review and editing, and resources. **Yurika Imai**: investigation, writing – review and editing, and resources. **Yuko Takahashi**: investigation, writing – review and editing, and resources. **Shizukiyo Ishikawa**: methodology, software, validation, formal analysis, supervision, and writing – review and editing. **Mina Morino**: investigation, resources, and writing – review and editing. **Hitomi Kashima**: investigation, resources, and writing – review and editing. **Takaya Miura**: investigation, writing – review and editing, and resources. **Takeshi Uehara**: investigation, writing – review and editing, and resources. **Hiroyuki Miyatani**: investigation, writing – review and editing, supervision, and resources. **Hirosato Mashima**: methodology, investigation, writing – review and editing, resources, supervision, conceptualization, and project administration. **Masanari Sekine**: investigation, writing – review and editing, and resources. **Takehiro Ishii**: investigation, writing – review and editing, and resources. **Takeharu Asano**: investigation, writing – review and editing, and resources. **Makiko Mieno**: methodology, writing – review and editing, validation, software, formal analysis, and supervision. **Haruka Otake**: investigation, writing – review and editing, and resources.

## Funding

The authors have nothing to report.

## Ethics Statement

Approval of the research protocol by an Institutional Review Board: This study was approved by the Ethics Committee of Jichi Medical University Saitama Medical Center (Approval No: Rin‐S24‐040).

## Consent

Informed consent was obtained using the opt‐out approach.

## Conflicts of Interest

The authors declare no conflicts of interest.
